# Xanthohumol-Enriched Beer Does Not Exert Antitumorigenic Effects on HeLa Cell Line In Vivo

**DOI:** 10.3390/molecules28031070

**Published:** 2023-01-20

**Authors:** Anna Júlia Éliás, Lajos Balogh, Tomáš Brányik, Erzsébet Mák, Éva Csajbókné Csobod, Márta Veresné Bálint, Csilla Benedek

**Affiliations:** 1 Doctoral School of Health Sciences, Semmelweis University, 1085 Budapest, Hungary; 2 NNK SSFO National Public Health Center, 1097 Budapest, Hungary; 3 Department of Biotechnology, University of Chemistry and Technology Prague, 166 28 Prague, Czech Republic; 4 Research Institute of Brewing and Malting, 120 00 Prague, Czech Republic; 5 Department of Dietetics and Nutritional Sciences, Faculty of Health Sciences, Semmelweis University, 1088 Budapest, Hungary

**Keywords:** neoplasms, beer, ethyl alcohol, xanthohumol, flavonoids, antioxidant

## Abstract

Xanthohumol is a hop-derived flavonoid that has been widely examined for its health-protecting and antitumorigenic properties, but not yet in a natural beer matrix. The aim of the study was to investigate the antitumorigenic potential of a xanthohumol-enriched beer in vivo. Four groups of 4 × 10 nude mice were formed. Following the injection of HeLa tumorigenic cell lines, the treatment groups were administered a xanthohumol supplementation for 100 days, either dissolved in beer or in an ethanolic solution with the same alcohol strength as beer. The control groups received un-supplemented material. The terminal tumor masses, liver weights, and plasma antioxidant capacities (FRAP and ABTS methods) were measured. For the statistical analysis, a two-way ANOVA test was performed (*p* < 0.05). There were no statistically significant differences in tumor size between the groups. Xanthohumol did not induce higher levels of plasma antioxidant capacity, neither in beer nor in the water–ethanol matrix. The terminal liver weights were significantly higher in the control group receiving the unsupplemented ethanol solution. Xanthohumol dissolved in beer or in the water–alcohol matrix did not have a protective effect on tumor growth, nor did it have a positive effect on plasma antioxidant capacity either. However, beer with added xanthohumol had a less harmful effect on the liver compared to the supplemented water–ethanol solution. Our results indicate the possible negative countereffect of ethanol; however, further investigations are needed.

## 1. Introduction

The prevention and treatment of cancer is one of the most important topics in medical research. Its spread and burden on society are significant, especially in Western countries. In addition to genetics and environmental factors, nutrition and lifestyle play a prominent role in the development of the disease. The harmful effects of red meat consumption, obesity, or a low-fiber, high-salt, high-fat diet are often highlighted, but the protective role of various phytonutrients, vitamins, or a balanced energy intake is also important to mention.

The antitumor activity of xanthohumol (XN) from hops (*Humulus lupulus* (Cannabinaceae)) has been demonstrated in previous studies, both in vitro and in vivo [1,2]. Several studies performed on mice reported decreased tumor growth and smaller terminal tumor sizes as a result of XN treatment [3,4,5,6,7,8]. The outstanding anticarcinogenic properties of XN, a substance belonging to the family of polyphenols, can be partly explained by its lipophilic antioxidant properties [9,10]. Nevertheless, it was demonstrated that xanthohumol, despite having a strong antioxidant activity, also causes apoptosis in a number of neoplastic cell lines by significantly increasing the formation of free radicals [11]. Although XN is present in significant amounts in hops, its concentration decreases markedly during brewing, despite numerous initiatives to increase the natural amount of XN in beer [12,13,14,15]. However, since beer contains a variety of other polyphenolic compounds [16,17], it is also regarded as a significant source of dietary antioxidants. Polyphenols are absorbed in the gastrointestinal tract and can be detected in the blood, improving thus the plasma antioxidant status, as demonstrated by several other studies on food [18] and beer antioxidants [19].

In spite of its health-protecting ingredients, beer also carries the carcinogenic effects of ethanol [20]. On the other hand, it can be assumed that the endogenous polyphenols in beer and its further fortification with XN may have a beneficial protective effect not only against carcinogenicity, but also against the development of fatty liver. The fibrogenesis-inhibiting and anti-inflammatory effects of pure XN on the liver have been reported under in vitro conditions. XN inhibits the activation of hepatic stellate cells, the primary cells driving fibrogenesis, and induces apoptosis in those that were already activated [21]. Furthermore, several in vivo studies proved that beers are less damaging to the liver than ethanol [22,23]. Further research revealed that beers containing no hops were as harmful as ethanol, whereas the traditional variety had a less harmful effect on the liver parameters studied [24]. It is therefore expected that beer with XN additives will also reduce the development of fatty liver when compared to a plain water–ethanol solution.

Our study has several new aspects compared to the ones previously reported. The study material was injected intraperitoneally or intragastrically in the majority of the previous animal studies investigating the antitumorigenic effect of XN. In our case, the mice ingested the substance orally by mixing it into their drinking fluid. We consider that the real novelty of the present research is that the previous literature has not addressed the impact of the beer matrix as a carrier on the effects of XN. Thus, the function of XN in its natural medium, beer, has not been described so far. The question is whether and to what extent the positive effects of XN will still be observable in this matrix. In addition, due to the natural alcohol content of beer, a new aspect was included in this study. This is an important aspect for future dietary considerations, as the merits of a new functional product (i.e., an XN-enriched beer) should be assessed by estimating both the harmful and protective factors. Therefore, it is necessary to clarify to what extent the beneficial properties of polyphenols and other bioactive substances in beer—which are otherwise anticarcinogenic [25]—can counterbalance the negative effects of alcohol. Newly published studies suggest that even low levels of alcohol consumption increase the risk of mortality, particularly caused by tumors; therefore, this question should not be ignored [26].

The aim of the present research is to evaluate the anticarcinogenic and liver-protective potential of beer containing an effective oral dose of XN [27], compared to the same dose administered in an ethanolic solution and to the same matrices without XN supplementation. The study was carried out by applying HeLa-cell tumor xenografts in immunodeficient nude mice, as this is (along with other immunodeficient rodents as experimental subjects) one of the most frequently used models in biomedical research [28]. The use of HeLa cells in our experiment investigating the antitumorigenic effects of xanthohumol in a beer matrix was also supported by the evidence that, in earlier in vitro studies, antioxidants from natural products (extracts from *Quercus resinosa* and *Aronia* spp.) proved to show anticancer activity in HeLa tumor cell cultures [29,30].

## 2. Results

Based on observations and measurements, each animal had developed tumors by the end of the 100 days of treatment. This was confirmed by the histopathological analysis as well. According to the report, the histological examination revealed a section of a solid epithelial tumor with extensive necrosis infiltrating the surrounding tissue. The tumor cells were characterized by marked anisokaryosis, and their nuclei were large, rounded or oval, heterochromatic, and contained large, prominent nucleoli. The cytoplasm was small to medium in volume, more or less vacuolated. The number of mitotic figures exceeded 30 mitoses/10 NNL (non-neoplastic lesions). Atypical mitoses and some giant-cell figures were also observed. The diagnosis was a high-grade, immature, solid carcinoma. There were no visible histological differences observed between the samples of any of the groups.

### 2.1. Terminal Tumor Weight

**Figure 1:**
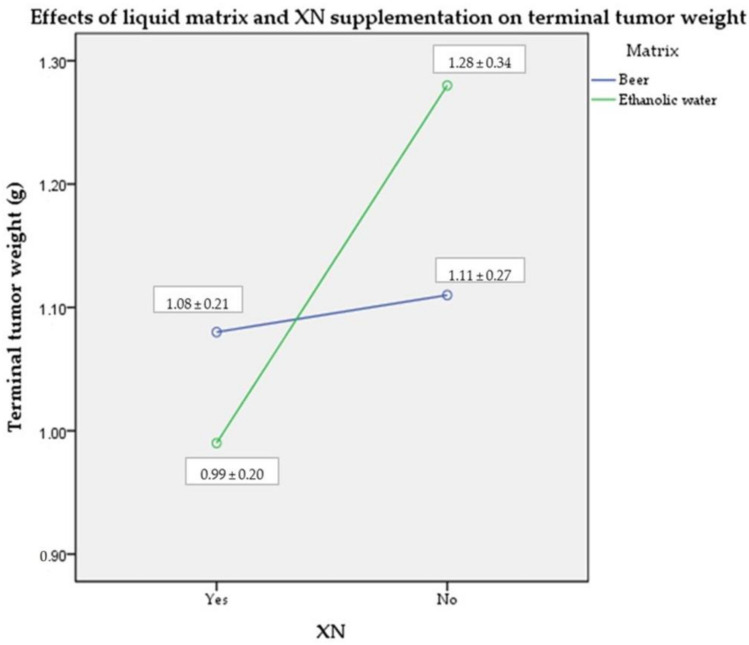
Mean (± SD) terminal tumor mass of mice per treatment group (*n* = 10). Abbreviations: XN—xanthohumol; SD—standard deviation.

The tumor masses were measured on the day of euthanasia. Although XN supplementation resulted in slightly smaller tumor weights with a medium effect size, these differences were not statistically significant (η_p_^2^ = 0.094; *p* > 0.05). The liquid matrix itself had only a small effect on the results (η_p_^2^ = 0.006; *p* > 0.05); however, the combined effect of the ethanolic matrix and the XN supplementation had a medium effect size, with no statistical significance (η_p_^2^ = 0.064; *p* > 0.05) (Figure 1).

### 2.2. Plasma Antioxidant Capacity by FRAP Assay

**Figure 2:**
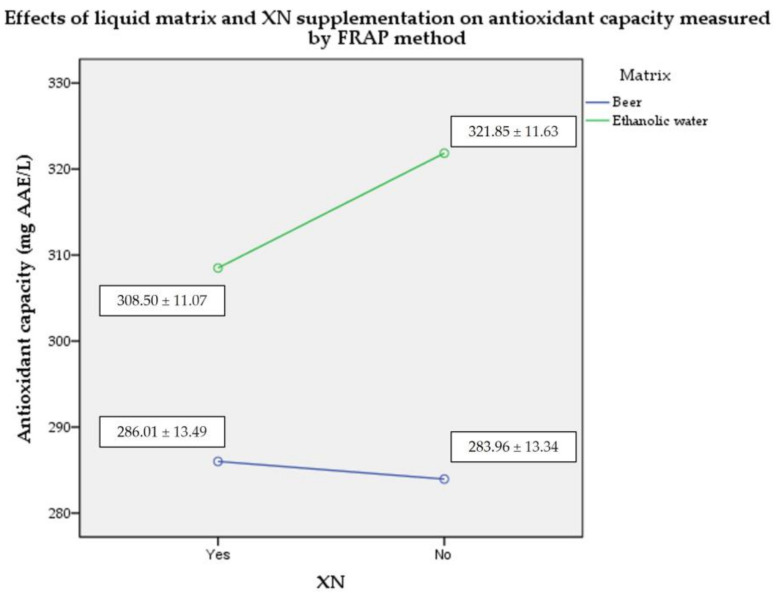
Mean (± SD) plasma antioxidant capacity values measured by FRAP method (*n* = 5). Abbreviations: AAE—ascorbic acid equivalent; FRAP—ferric reducing ability of plasma; XN—xanthohumol; SD—standard deviation.

When measured by FRAP, the ethanol-based groups showed more favorable results in terms of serum antioxidant capacity compared to the beer-based groups (large effect size of liquid matrix; η_p_^2^ = 0.648; *p* < 0.05). However, there was no significant difference between the XN-fortified and unfortified variations (medium effect size of XN supplementation and combined effect with ethanolic matrix; η_p_^2^ = 0.61 and 0.107; *p* > 0.05) (Figure 2).

### 2.3. Plasma Antioxidant Capacity by ABTS Assay

**Figure 3:**
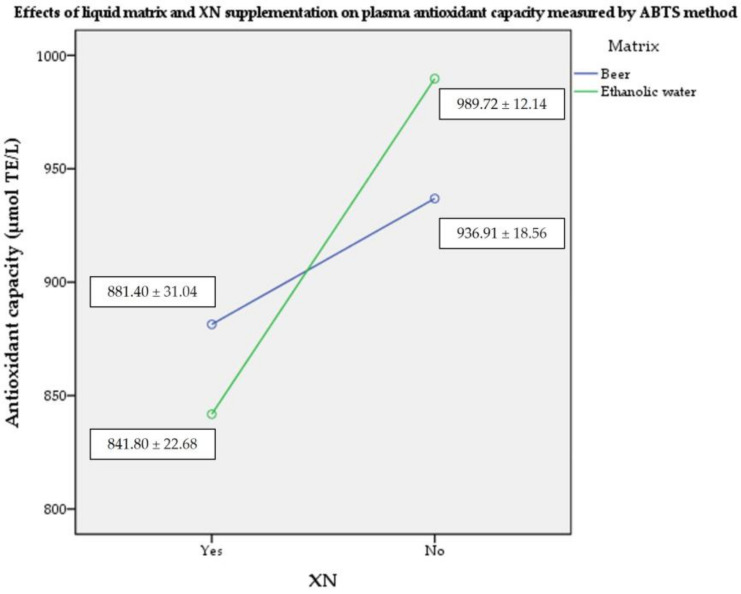
Mean (± SD) plasma antioxidant capacity values measured by ABTS method (*n* = 4). Abbreviations: TE—Trolox equivalents; ABTS—2,2′-azino-bis(3-ethylbenzothiazoline-6-sulfonic acid; XN—xanthohumol; SD—standard deviation.

When measured by ABTS assay, both the consumption of unfortified beer and of ethanol resulted in significantly higher levels of plasma antioxidant capacity compared to the fortified beverages (large effect size of no supplementation; η_p_^2^ = 0.885; *p* < 0.05). The latter also resulted in a higher capacity when compared to the consumption of unfortified beer (large size of joint effect; η_p_^2^ = 0.615; *p* < 0.05). The liquid matrix itself had a small effect on the results (η_p_^2^ = 0.032.; *p* > 0.05) (Figure 3).

### 2.4. Terminal Liver Weight

**Figure 4:**
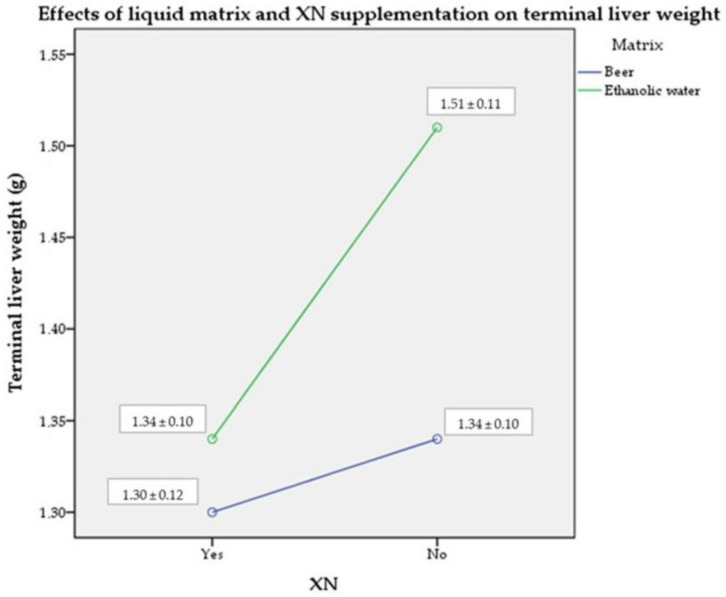
Mean (± SD) terminal liver mass of mice by treatment group (*n* = 10). Abbreviations: XN—xanthohumol; SD—standard deviation.

Liver weights were measured on the day of euthanasia. The mice receiving unfortified ethanol had significantly higher liver weights compared to the other groups (medium combined size effect of the absence of XN fortification and the ethanolic liquid η_p_^2^ = 0.096; *p* > 0.05). The XN supplementation and the liquid matrix had a large effect size on their own (η_p_^2^ = 0.217; *p* < 0.05) (Figure 4). These results show a favorable impact of both the beer matrix and the XN fortification on terminal liver weight.

## 3. Discussion

The results obtained on tumor mass may be attributed to several factors. Previous in vivo studies that have reported a protective effect of XN have generally used higher concentrations of XN. On a body-weight basis, experimental animals received as much as 2–5–15–30 mg/kg XN per day; however, in these cases, the substance was administered intraperitoneally or intragastrically, which made it easier to deliver a higher dose [3,5,8]. In contrast, in our experiment, the daily dose was 0.3–0.365 mg/kg (depending on the treatment group). This was based on a study with similar XN doses [31], where a positive effect was clearly induced by similar or even lower doses (0.071 mg/kg/day and 0.213 mg/kg/day of XN). However, one of the main differences was the presence of an alcoholic medium, which in our case was more than six times higher than the exposure used in the abovementioned study by Ferk et al. [31]. Since several other studies [3,4,5,6,8] have shown that XN can play a protective role in carcinogenesis, we hypothesize that the combination of lower XN content and the presence of alcohol may explain our results. It is therefore of paramount importance to consider the influential role of alcohol. Alcohol-induced tumorigenic and metastatic lesions have also been explained by the immunosuppressive effect of alcohol exposure. Inappropriate DNA methylation may be an important epigenetic factor contributing to the pathogenic effects [32,33,34]. Most previous studies have used only minimal amounts of ethanol for XN dissolution, and thus virtually excluded its influence. Thus, the effect of alcohol as a carrier matrix of XN has not been investigated previously. It can be assumed that, at the levels used in our study, XN can only partially counteract the adverse factors of the alcoholic medium.

When measuring in vitro plasma antioxidant capacity, beer was expected to induce a higher plasma antioxidant capacity compared to the water–ethanol solution due to its own antioxidant properties; however, this hypothesis has not been confirmed. There are several possible reasons for the phenomenon observed. Firstly, ethanol, while mostly known for its negative physiological effects, is also known for its antioxidant properties [35]. These are assumed to contribute to the final in vitro results obtained by us. In addition, the literature describes the ability of plasma proteins to form complexes with polyphenols [36]. Thus, they can ‘mask’ the presence of polyphenols, resulting in so-called ‘masked’ measurement results, which can provide a plausible explanation for our results. Several studies have highlighted that the antioxidant capacity of certain polyphenol-containing foods also depends on the presence of other substances (e.g., proteins, polysaccharides, etc.) with which they interact [37,38]. Blood plasma itself has antioxidant properties, as it contains many free-radical-scavenging endogenous elements. However, when these interact with other antioxidant compounds, the effects may not always be additive, and may even be antagonistic. For example, amino acids and polysaccharides can significantly affect the results obtained, so that the specific levels depend not only on the amount of antioxidants present, but also on several characteristics of the matrix [36]. The lower results obtained for the fortified samples may be attributed to the lipophilic character of XN, which does not exert its antioxidant capacity in the hydrophilic media used in the in vitro assays applied.

As our research objective was essentially to observe tumor growth, we did not investigate more detailed markers or functions of the liver beyond its weight. The results were consistent with our hypothesis that ethanol increases the risk of fatty liver. At the same time, our measurements confirmed the protective effect of XN: the consumption of XN-containing beverages resulted in a lower liver mass compared to the unfortified ethanol control.

In conclusion, we believe that the importance of the research on XN could grow even further in the future. Based on our results, further research could provide a more accurate picture of the underlying mechanisms. On the other hand, during product development, any circumstances that may reduce the health-protective value of the product should be taken into account. Accordingly, three main aspects for further research are recommended: (1) higher XN concentration; (2) reduced alcohol content; (3) further investigation of human applications.

### Limitations of the Study

The results of preclinical animal studies are not fully applicable to the human body, so further studies are needed to substantiate the findings. Our heterotopic model does not provide the same microenvironment for the cells as if they were in the original organ; therefore, differences in tumor behavior may occur.

Although the XN content of the beer we used for the experiment (Dreher Classic pale ale) was in accordance with the international literature, we can only conclude that this certain product does not alter the effect of xanthohumol on tumor growth. There are hundreds of beer types with various compositions, so there is a chance (although a limited one) that some types of beer can positively or negatively modulate the effect of xanthohumol on tumor growth.

## 4. Materials and Methods

To maximize the quality and reliability of this published research, we used the ARRIVE guidelines (Animal Research: Reporting of In Vivo Experiments) [39]. The experiments were performed at the Department of Radiation Oncology of the National Centre for Public Health, Hungary. Mice were procured, fed, followed-up on, and euthanized under appropriate and standard conditions. The experiment was conducted according to the standard guidelines for tumorigenicity testing [40]. We have submitted our ethical approval request to the Institutional Research Ethics Committee. The authorization number is PE/EA/1412-6/2016. Sample and study solution preparation and in vitro plasma antioxidant-capacity measurements were performed at the Laboratory of Food Chemistry, Faculty of Health Sciences, Semmelweis University, Hungary. The XN content of the original beer was measured at the University of Chemistry and Technology in Prague. Chemicals were analytical-grade and provided by Sigma Aldrich.

### 4.1. Analytical Procedure of XN Content Measurement

Sample preparation was performed by solid phase extraction (SPE). For this purpose, the extraction charge was first conditioned with 10 mL methanol followed by 10 mL deionized acidified water (H_3_PO_4_ (100:0.2, *v/v*)). Then, 25 mL of beer was de-foamed and acidified with 0.1 mL of H_3_PO_4_. Contaminants were removed by solvent rinsing with methanol + water + H_3_PO_4_ (20:80:0.2, *v/v/v/v*), followed by eluting the target component retained on the column with acidified methanol (0.2, *v/v* H_3_PO_4_). The analysis was performed by using high-performance liquid chromatography (HPLC). Mobile phases: A—deionized water (+0.05% formic acid), B—acetonitrile (+0.05% formic acid). Gradients: from 0 to 42 min, mobile phase B increased linearly from 35% to 95%. Quantification was based on standard calibration. The detailed procedure is presented in Supplementary File S1 (Supplementary Materials).

According to our measurement, the endogenous XN content of the beer used was 0.16 mg/L, which is in line with the literature [41].

### 4.2. Study Subjects and Study Design

As heterotopic models, 4 × 10 BALB/c nude immunosuppressed mice (without thymus), each of them 4–7 weeks old, were investigated. Since they were identical species, no inclusion or exclusion criteria were set up. The 40 specimens were randomly divided into 4 × 10 groups. After in vitro cultivation, 10⁷ living HeLa cells (ATCC No.: CRM-CCL-2, BSL 2) were suspended in 100 microliters of physiological saline. This tumor-cell suspension was injected into the left-thigh muscles of tumor-free mice (10⁷ cells/0.1 mL, intramuscular), according to the protocol, which normally results in 100% tumor development. This was taken as evidence based on previous experience. The intervention started immediately after the cell implantation. The animals consumed the study solution orally for 100 days according to their group allocation. Mice were controlled visually and by finger palpation to recognize the tumor growth 3 times a week (Monday-Wednesday-Friday schedule). After the treatment period, each anesthetized mice underwent final samplings that included body- and tumor-weight measurement, blood sampling, and gross pathology. The inoculated tumors were removed from the affected legs, scaled and immediately stored in 4% buffered formalin solution. Histopathological evaluation (hematoxylin and eosin staining) was carried out not later than 4 weeks post-removal by a pathologist expert. The antioxidant capacity of the animals’ plasma was also analyzed. None of the animals died before the end of the experimental period, and all data were used in the statistical analysis.

### 4.3. Study Sample Preparation

**Figure 5:**
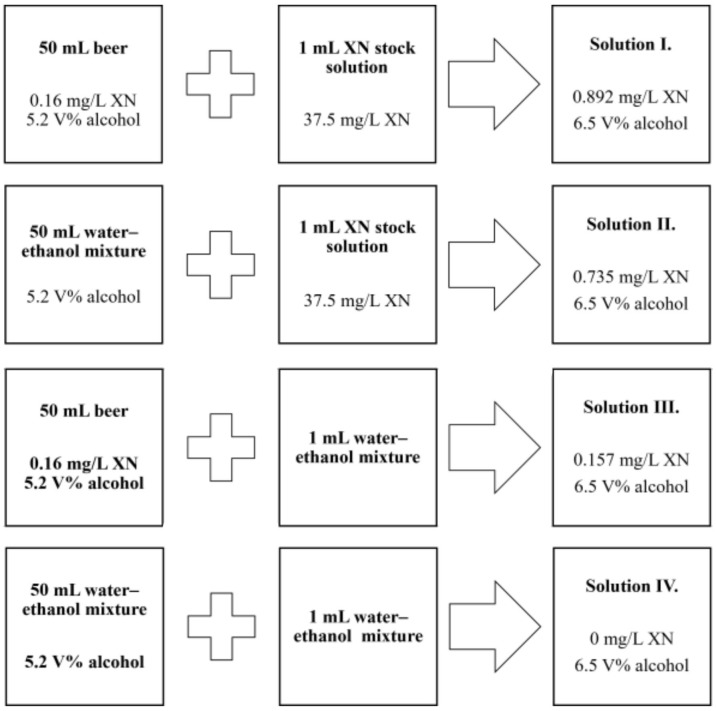
Preparation and characteristics of study solutions.

For the XN stock solution, 400 mL of a 50% aqueous alcohol mixture was prepared to ensure proper solubility of XN. In this mixture, 15 mg of chemically pure XN of hop origin (Sigma Aldrich) was dissolved. The stock solution was divided into 400 × 1 mL Eppendorf tubes and stored frozen at −18 °C. Commercially available beer (Dreher Classic pale ale, 5.2%) bottled from the same production batch (12 × 0.5 L) was used for the experiment. During the intervention period, the drinking solutions were prepared daily. The steps for preparing the solutions, and the XN and alcohol contents for each sample, are shown in Figure 5. These amounts of liquids were used for 5 mice/day. The above drinks were given to the experimental animals ad libitum, according to the experimental design. Liquids were fully consumed in all groups every day. It is important to point out that the alcohol concentration of all the drinks from each of the 4 groups was the same (final concentration 6.5 V%). The concentration achieved by enrichment (neglecting the natural XN content of the beer) was selected as an effective oral dose based on the previous literature [27]. Based on the recommendations for use and storage of XN [42,43], the drinking samples were replaced daily by fresh solutions. Mice consumed their usual standard rodent food, ad libitum, throughout the experiment.

### 4.4. Measurement of Antioxidant Capacity

The antioxidant capacity of the mice blood plasma was determined by two in vitro methods. Since the amount of sample per mouse is extremely small, pooled plasma samples were used per group. For ferric reducing antioxidant power (FRAP) measurements, the method developed by Benzie and Strain [44] was adopted and performed with 5 replicates. The 2,2′-azino-bis(3-ethylbenzothiazoline-6-sulfonic acid) (ABTS) method was performed according to the method described by Re et al. [45] with 4 replicates.

#### 4.4.1. FRAP Method

To carry out the FRAP measurement, 0.54 g FeCl_3_ was dissolved in distilled water in a 100 mL flask and then adjusted to the mark. In another flask, 0.3123 g TPTZ (2,4,6-tripyridyl-S-triazine) was dissolved in a diluted hydrochloric acid solution of 40 mmol/L, then this was also adjusted to 100 mL. The two solutions were mixed and diluted to 1 L with pH 3.6 (300 mM Na-acetate) buffer (FRAP stock solution). A total of 50 μL of the undiluted plasma samples was pipetted into test tubes and 7.5 mL of FRAP stock solution was added to each sample. The test tubes were incubated at 30 °C for 30 min. The absorbance was measured at λ = 593 nm; results were expressed based on calibration with ascorbic acid (mg ascorbic acid equivalents/liter beer, calibration range: 0–1250 mg ascorbic acid/liter). Measurements were performed using a Thermo Helios-α (Thermo Spectronic, Cambridge, UK) spectrophotometer.

#### 4.4.2. ABTS Method

For the ABTS measurement, an aqueous solution of the ABTS compound was prepared at a concentration of 7 mM. This stock solution was reacted with 2.45 mM potassium persulphate and allowed to stand for 16 h in the dark at room temperature in order to develop the ABTS·^+^ stock solution. From the above stock solution, 2 mL was diluted with 100 mL ethanol to give an absorbance of 0.7 at λ = 734 nm. A total of 2 mL of each of the pooled plasma samples was measured into 10 mL volumetric flasks and brought to the mark with distilled water, thus creating a fivefold dilution. From these samples, 0.1 mL was pipetted into the tubes and 3.9 mL of ABTS^+^ solution was added. The solutions were mixed and poured into cuvettes after standing for 5 min. Measurements were carried out at λ = 734 nm with Trolox as calibrating substance (calibration range: 0–1250 μmol Trolox/liter). Results were expressed as μmol Trolox equivalents/liter beer. A Thermo Helios-α (Thermo Spectronic, Cambridge, England) spectrophotometer was used for the measurements.

### 4.5. Statistical Analysis

Microsoft Office 365 Excel, XLSTAT, and IBM SPSS Statistics 20 software were used for statistical analysis. The impact of treatment on the parameters was evaluated by a two-factor ANOVA test (analysis of variance). The two factors investigated were the liquid matrix (beer or water–ethanol) and the XN supplementation (yes or no). Post hoc tests were not performed because there were fewer than three groups for each investigated factor. Adjustment for multiple comparisons were performed by Bonferroni test. Differences were considered significant when *p* < 0.05 was reached. Normality was tested by Shapiro–Wilk test, and homogeneity of variance by Levene test. Effect sizes were measured by partial eta-squared (η_p_^2^) and categorized as follows: 0.01—small effect size; 0.06—medium effect size; 0.14 or higher—large effect size [46].

## 5. Conclusions

Xanthohumol administered to mice orally in a water–alcohol-based matrix did not have a direct protective effect on tumor growth (induced by the injection of HeLa tumorigenic cell lines) and did not have a positive effect on plasma antioxidant capacity either. However, xanthohumol administered in the form of a beer solution had a less harmful effect on the liver compared to the water–ethanol solution with the same alcohol and xanthohumol concentrations. Our results indicate the possible negative countereffect of ethanol; however, further investigations are needed.

Our findings suggest several directions for further research. Firstly, our experiments indicate that it is worthwhile to investigate the effects of a higher XN concentration, as XN has been proven to have a good safety profile [47,48]. Based on our results, another important aspect could be the reduction in alcohol content. This would lower the adverse effects of ethanol observed in this study. However, the weak water solubility of xanthohumol may be a limiting factor.

In conclusion, the importance of further research on XN as a potent anticarcinogenic agent may increase in the future. In the development of a XN-based functional health product, preliminary in vivo experimental experience is essential to unravel the factors that may result in the maximum health benefit of the product.

## Data Availability

The data presented in this study are available in this article and the Supplementary Materials.

## Supplementary Materials

The following supporting information can be downloaded at: https://www.mdpi.com/article/10.3390/molecules28031070/s1.
