# Supplementary material for: Xanthohumol-Enriched Beer Does Not Exert Antitumorigenic Effects on HeLa Cell Line In Vivo
Source: Molecules. 2023 Jan 20;28(3):1070. doi: 10.3390/molecules28031070 (PMC9918897; doi:10.3390/molecules28031070)
Supplement: Supplementary file 1 [file molecules-28-01070-s001.zip › molecules-2077292-supplementary.pdf]

Supplementary File S1. Detailed description of the method analytical procedure for measuring the XN content of the beer tested

Analytical procedure

Xanthohumol was extracted from beer using solid phase extraction column, SPE, (Strata C18-E, 500 mg/6 mL, Phenomenex). The SPE column was equilibrated by 10 mL of methanol and subsequently by 10 mL of acidified water (solution No. 1). 25 mL of beer sample was defoamed by sonification and acidified by 0.1 mL of H_3PO_4 and then the sample was applied on conditioned SPE column. Using of 10 mL of solution No. 2, more polar impurities were discarded. Analytes were eluted by solution No. 3 into final volume 2.5 mL after 5 minutes of air drying-up.

Chemicals

Reference standard xanthohumol was from AAPIN Chemicals Limited (UK).

Calibration solutions: stock standard solution was prepared by weighting out 0.2–1.0 mg ± 0.1 mg exactly of each compound into a 5 mL volumetric flask and made the contents up to volume with gradient grade methanol (Merck) at 20 °C. Calibration solutions were prepared by dilution of stock standard 10×, 50×, 100× and 1000 × into the 10 mL beakers in methanol. Flasks were stoppered and mixed by inversion at least four times.

Other solutions were prepared using deionized water (Millipore. TOC < 5 ppb), methanol analytical-reagent grade (Merck) and 85 % H_3PO_4 analytical-reagent grade (Merck) and acetone analytical – reagent grade (Lachner).

For extraction of beers:

1. deionized water acidified H_3PO_4 (100:0.2, v/v)

2. methanol + water +H_3PO_4 (20:80:0.2, v/v/v)

3. acidified methanol H_3PO_4 (100 : 0.2, v/v)

For other details see https://www.kvasnyprumysl.cz/pdfs/kpr/2013/02/02.pdf

HPLC analysis

Mobile phase:

A – deaerated demineralized water (0.05 % wt. formic acid)

B – deaerated acetonitrile (0.05 % wt. formic acid)

Gradient (Table 1):

From 0 to 42 minutes the proportion of mobile phase B linearly increased from 35 % to 95 % vol.

Table 1. Gradient of mobile phases (% vol.)

Time (min)	A	B
**0**	65	35
**40**	38	62
**42**	5	95
**47**	5	95

Temperature: 30 °C

Flow rate: 0,8 ml/min

Detector: PDA (Absorbance of xanthohumolu was detected at 370 nm).

Injection: 10 μL

Analytical equipment: HPLC chromatograph 1100 Series (Agilent, USA), PDA detektor (Agilent, USA), Column ECLIPSE XDB-C18, 5 μm, 4.6 x 150 mm (Agilent, USA)

Supplementary Table S1. Individual terminal body weight and tumour masses of the mice per group

Group	TBW (g)	TTW (g)	TLW (g)
A1	27.4	1.4	1.4
A2	24.6	0.9	1.2
A3	26.5	0.8	1.4
A4	25.8	0.9	1.3
A5	26.4	1.1	1.3
A6	25.3	1.2	1.2
A7	24.9	0.9	1.1
A8	26.4	1.4	1.3
A9	27	1.2	1.5
A10	26.4	1	1.3
B1	25.4	1.2	1.3
B2	26.4	1	1.4
B3	24.8	1.1	1.3
B4	24.5	1.1	1.3
B5	25.3	1.3	1.4
B6	26.2	0.7	1.5
B7	25.8	1	1.4
B8	25	0.7	1.2
B9	26.2	1	1.4
B10	24.9	0.8	1.2
C1	25.4	1.2	1.3
C2	26.1	1	1.4
C3	25.7	0.8	1.3
C4	24	1	1.3
C5	24.8	0.7	1.4
C6	25.6	0.9	1.5
C7	24.5	1.5	1.4
C8	26.1	1.4	1.2
C9	25.4	1.2	1.4
C10	24.7	1.4	1.2
D1	24.6	1.3	1.4
D2	25.4	1.2	1.5
D3	25.8	0.8	1.6
D4	25.2	1.2	1.6
D5	24.4	1.7	1.4
D6	23.8	1.8	1.4
D7	24.6	1.5	1.5
D8	24.4	1.1	1.4
D9	25.2	1.4	1.6
D10	26	0.8	1.7

Group A: beer+xanthohumol; Group B: ethanolic water+xanthohumol; Group C: beer; Group D: ethanolic water
Abbreviations: TBW: terminal body weight; TTW: terminal tumour weight; TLW: terminal liver weight

Supplementary Table S2. Replicate results of plasma antioxidant capacity measured by FRAP method in each group

Group	Plasma antioxidant capacity FRAP (mg ASE/L)				
	Replicate measurements				
Beer+xanthohumol	265.83	298.03	297.38	288.94	279.85
Ethanolic water+xanthohumol	291.80	317.51	316.99	302.71	313.48
Beer	275.96	278.55	304.52	289.59	271.15
Ethanolic water	304.78	327.64	336.08	322.57	318.16

Supplementary Table S3. Replicate results of plasma antioxidant capacity measured by ABTS method in each group

Group	Plasma antioxidant capacity ABTS (μmol TE/L)			
	Replicate measurements			
Beer+xanthohumol	862.87	864.11	917.23	
Ethanolic water+xanthohumol	832.04	837.46	823.06	874.64
Beer	914.44	929.01	950.38	953.79
Ethanolic water	986.78	998.71	973.77	999.64

Supplementary Table S4. Descriptive statistics of the measured parameters

Descriptive Statistics

Liquid matrix		Tumor weight (g) Mean	Tumor weight (g) Std. Deviation	Tumor weight (g) N	Liver weight (g) Mean	Liver weight (g) Std. Deviation	Liver weight (g) N	Antioxidant capacity mg/L (FRAP) Mean	Antioxidant capacity mg/L (FRAP) Std. Deviation	Antioxidant capacity mg/L (FRAP) N	Antioxidant capacity µmol/L (ABTS) Mean	Antioxidant capacity µmol/L (ABTS) Std. Deviation	Antioxidant capacity µmol/L (ABTS) N
Beer	XN Yes	1.0800	.21499	10	1.3000	.11547	10	286.0077	13.48696	5	881.4021	31.03761	3
	XN No	1.1100	.27264	10	1.3400	.09661	10	283.9562	13.34022	5	936.9056	18.56051	4
	Total	1.0950	.23946	20	1.3200	.10563	20	284.9820	12.69278	10	913.1184	37.06129	7
Ethanolic water	XN Yes	.9900	.20248	10	1.3400	.09661	10	308.4970	11.07410	5	841.8011	22.68329	4
	XN no	1.2800	.33599	10	1.5100	.11005	10	321.8451	11.63283	5	989.7243	12.14046	4
	Total	1.1350	.30826	20	1.4250	.13328	20	315.1711	12.81173	10	915.7627	80.84228	8
Total	XN Yes	1.0350	.20844	20	1.3200	.10563	20	297.2524	16.60841	10	858.7730	32.03821	7
	XN No	1.1950	.31031	20	1.4250	.13328	20	302.9007	23.19500	10	963.3149	31.74737	8
	Total	1.1150	.27321	40	1.3725	.13006	40	300.0765	19.84697	20	914.5287	62.11490	15

Supplementary Table S5. Testo of between-subject effects of tumor weight

Tests of Between-Subjects Effects

Dependent Variable: tumor weight

Source	Type III Sum of Squares	df	Mean Square	F	Sig.	Partial Eta Squared
Corrected Model	.441[a]	3	.147	2.143	.112	.151
Intercept	49.729	1	49.729	724.795	.000	.953
Liquid matrix	.016	1	.016	.233	.632	.006
Xanthohumol	.256	1	.256	3.731	.061	.094
Liquid matrix * Xanthohumol	.169	1	.169	2.463	.125	.064
Error	2.470	36	.069			
Total	52.640	40				
Corrected Total	2.911	39				

a. R Squared = .151 (Adjusted R Squared = .081)

Supplementary Table S6. Testo of between-subject effects of Antioxidant capacity measured by FRAP

Tests of Between-Subjects Effects

Dependent Variable: FRAP

Source	Type III Sum of Squares	df	Mean Square	F	Sig.	Partial Eta Squared
Corrected Model	5012.871[a]	3	1670.957	10.818	.000	.670
Intercept	1800918.326	1	1800918.326	11659.868	.000	.999
Xanthohumol	159.516	1	159.516	1.033	.325	.061
Liquid matrix	4556.916	1	4556.916	29.503	.000	.648
Xanthohumol * Liquid matrix	296.438	1	296.438	1.919	.185	.107
Error	2471.271	16	154.454			
Total	1808402.467	20				
Corrected Total	7484.142	19				

a. R Squared = .670 (Adjusted R Squared = .608)

Supplementary Table S7. Testo of between-subject effects of Antioxidant capacity measured by ABTS

Tests of Between-Subjects Effects

Dependent Variable: ABTS

Source	Type III Sum of Squares	df	Mean Square	F	Sig.	Partial Eta Squared
Corrected Model	49069.741[a]	3	16356.580	36.378	.000	.908
Intercept	12296567.589	1	12296567.589	27348.291	.000	1.000
Xanthohumol	38199.149	1	38199.149	84.957	.000	.885
Liquid matrix	161.265	1	161.265	.359	.561	.032
Xanthohumol * Liquid matrix	7884.376	1	7884.376	17.535	.002	.615
Error	4945.912	11	449.628			
Total	12599456.251	15				
Corrected Total	54015.653	14				

a. R Squared = .908 (Adjusted R Squared = .883)

Supplementary Table S8. Test of between-subject effects of Liver weights

Tests of Between-Subjects Effects

Dependent Variable: Liverweight

Source	Type III Sum of Squares	df	Mean Square	F	Sig.	Partial Eta Squared
Corrected Model	.263[a]	3	.088	7.942	.000	.398
Intercept	75.350	1	75.350	6832.768	.000	.995
Matrix	.110	1	.110	9.997	.003	.217
xn	.110	1	.110	9.997	.003	.217
Matrix * xn	.042	1	.042	3.831	.058	.096
Error	.397	36	.011			
Total	76.010	40				
Corrected Total	.660	39				

a. R Squared = .398 (Adjusted R Squared = .348)

Supplementary Figure S1. Calibration curve of xanthohumol

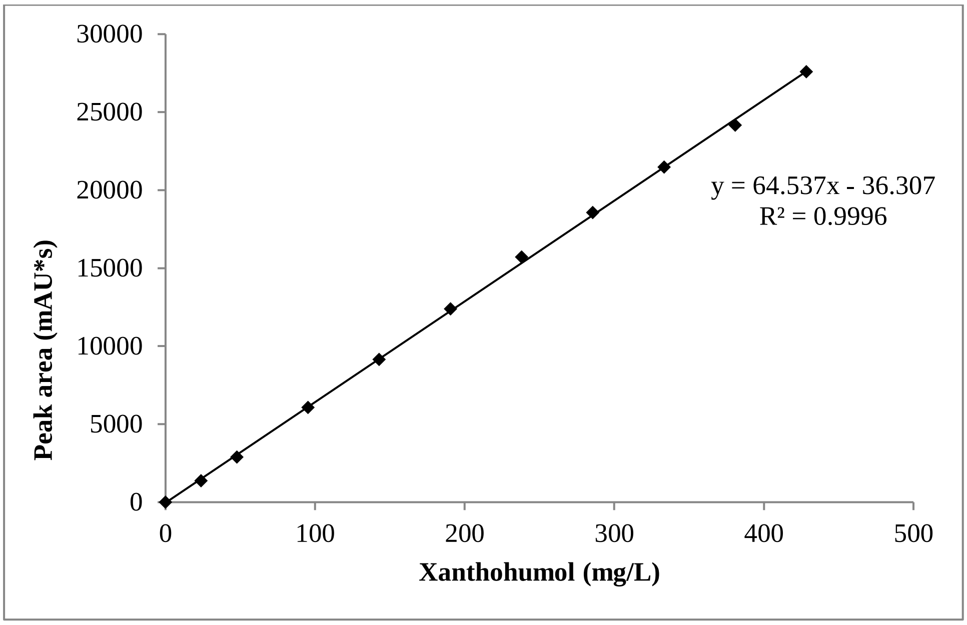

Retention time of xanthohumol 31.49 min.

Reproducibility of xanthohumol determination 2.53 % rel.
